# Docetaxel does not impair skeletal muscle force production in a murine model of cancer chemotherapy

**DOI:** 10.14814/phy2.13261

**Published:** 2017-06-05

**Authors:** Thomas Chaillou, Ashley McPeek, Johanna T. Lanner

**Affiliations:** ^1^ Department of Physiology and PharmacologyKarolinska InstitutetStockholmSweden; ^2^ School of Health SciencesÖrebro UniversityÖrebroSweden

**Keywords:** Acute and repeated treatments, disease, fatigue, muscle weakness, recovery

## Abstract

Chemotherapy drugs such as docetaxel are commonly used to treat cancer. Cancer patients treated with chemotherapy experience decreased physical fitness, muscle weakness and fatigue. To date, it is unclear whether these symptoms result only from cancer‐derived factors or from the combination of cancer disease and cancer treatments, such as chemotherapy. In this study, we aimed at determining the impact of chemotherapy *per se* on force production of hind limb muscles from healthy mice treated with docetaxel. We hypothesized that docetaxel will decrease maximal force, exacerbate the force decline during repeated contractions and impair recovery after fatiguing stimulations. We examined the function of soleus and extensor digitorum longus (EDL) muscles 24 h and 72 h after a single injection of docetaxel (acute treatment), and 7 days after the third weekly injection of docetaxel (repeated treatment). Docetaxel was administrated by intravenous injection (20 mg/kg) in female FVB/NRj mice and control mice were injected with saline solution. Our results show that neither acute nor repeated docetaxel treatment significantly alters force production during maximal contractions, repeated contractions or recovery. Only a tendency to decreased peak specific force was observed in soleus muscles 24 h after a single injection of docetaxel (−17%, *P *=* *0.13). In conclusion, docetaxel administered intravenously does not impair force production in hind limb muscles from healthy mice. It remains to be clarified whether docetaxel, or other chemotherapy drugs, affect muscle function in subjects with cancer and whether the side effects associated with chemotherapy (neurotoxicity, central fatigue, decreased physical activity, etc.) are responsible for the experienced muscle weakness and fatigue.

## Introduction

Fatigue syndrome, decreased physical fitness and muscle weakness are commonly reported complications for patients with cancer (Curt et al. 2000; Santiago‐Palma and Payne 2001; Perry et al. 2007). These debilitating symptoms can lead to a reduced quality of life (QoL) (Franc et al. 2014) and contribute to an increased mortality risk in patients with cancer (Adams et al. 2016). However, it is currently unclear whether these symptoms are only caused by cancer‐derived factors or by the combination of cancer disease and cancer treatments (surgery, radiotherapy, systemic therapy, etc.).

Chemotherapy is commonly used as a systemic therapy to treat cancer. Taxane‐derived chemotherapy agents are widely used in cancer treatment. These drugs disrupt microtubule function and inhibit the process of cell division, leading to the suppression of cancer cell growth. Docetaxel and paclitaxel are among the most commonly prescribed taxane‐derived cytotoxic drugs approved for the treatment of metastatic or locally advanced breast cancers, as well as in the adjuvant setting for operable node‐positive breast cancers (Martin et al. 2005; Nabholtz and Gligorov 2005). Both taxanes are also approved for treatment of prostate, gastric, head and neck, non‐small cell lung, and ovarian cancers (Joerger 2016). Docetaxel was developed with the aim of improving the features of paclitaxel and has contributed to improvements in cancer survival (Petrylak et al. 2004; Bria et al. 2006; van Cutsem et al. 2006). Despite docetaxel's efficacy as a cancer treatment, it causes a variety of side effects that can decrease the QoL of patients. The reported short‐ and long‐term side effects of docetaxel treatment vary between individuals, but the most common symptoms include diarrhea, stomatitis, nausea, dyspnea, fatigue, infection, and anemia (AL‐Batran et al. 2015).

Decreased physical fitness, muscle weakness and muscle fatigue have been reported in cancer patients treated with chemotherapy (Stone et al. 1999; Harrington et al. 2011). However, due to the complexity of each cancer diagnosis and the putative negative effects of the cancer treatment itself, it remains to be elucidated how chemotherapy *per se* affects muscle function. Several studies have investigated the effect of chemotherapy on muscle force production in mice. However, the majority of them used the chemotherapy drug doxorubicin and evaluated muscle function after a single intraperitoneal (IP) injection (Gilliam et al., 2009, Gilliam et al. 2011b, 2016). Specifically, it was shown that force production was decreased in both the respiratory muscle (diaphragm) and the hind limb muscle (extensor digitorum longus, EDL) after acute IP administration with doxorubicin (Gilliam et al. 2011b, Gilliam et al., 2009).

The dosage of docetaxel is patient‐specific but the treatment is usually administered intravenously and typically follows a weekly or 3‐weekly schedule (Hainsworth 2004). In this study, we aimed at determining whether docetaxel chemotherapy affects the force production of hind limb muscles in healthy mice after an acute treatment, and after a repeated treatment. We hypothesized that docetaxel will decrease maximal force, exacerbate the force decline during repeated contractions and impair recovery after fatiguing stimulations. To test our hypothesis, we examined the function of the soleus muscles (slow‐twitch, oxidative) and EDL muscles (fast‐twitch, glycolytic) 24 h and 72 h after a single intravenous (IV) injection of docetaxel (acute treatment), and 7 days after the third weekly IV injection of docetaxel (repeated treatment).

## Material and Methods

### Animals

Systemic administration of docetaxel was performed at Karolinska Institutet (Solna, Sweden) using 8‐ to 11‐wk old female mice on FVB/NRj strain background (Janvier Labs, France). This mouse strain was chosen because the animals have the same background as the transgenic mice commonly used to study breast cancer, the mice mammary tumor virus (MMTV) encoding the polyoma virus middle T antigen (PyMT) (FVB/N‐MMTV‐PyMT mice) (Cunha et al. 2015). All animal experiments were conducted according to the regulations of the Karolinska Institutet and were approved by the local laboratory animal ethics committee. These experiments comply with the Swedish Animal Welfare Act and the recommendations from Swedish authorities. The mice were housed in a temperature‐ and humidity‐controlled facility (12:12 h light:dark cycle, 22°C) with access to food and water ad libitum. The mice were euthanized by rapid neck disarticulation.

### Docetaxel treatment

Docetaxel (Taxotere, Actavis, Hafnarjordur, Iceland) was purchased in infusion solution of 20 mg/mL. The docetaxel solution was diluted in 0.9% saline solution to get a final concentration of 3 mg/mL. The animals received 20 mg/kg docetaxel by IV tail vein injection. IV injection was performed because this type of administration is the most commonly used in patients with cancer treated with chemotherapy. The amount of drug was based on a conversion factor derived from the relationship between body mass and body surface area of the animal (Nair and Jacob 2016), and was equivalent to 60 mg/m^2^. This dose is within the clinical dosing regimen observed in patients with breast cancer treated with docetaxel chemotherapeutic agent (Kongsted et al. 2015; Masuda et al. 2016; Meulendijks et al. 2016). Control (Ctrl) mice received by IV tail vein injection, the same volume of 0.9% saline solution. Animals (*N* = 4–6 per group) were randomly assigned to one of the experimental groups. For acute treatment, mice were sacrificed either 24 h (Docetaxel 24 h) or 72 h (Docetaxel 72 h) after the IV injection of docetaxel. For repeated treatment, animals were euthanized 7 days after the third weekly IV injection of docetaxel (Docetaxel 3 week).

### Skeletal muscle contractile function, fatigue, and recovery

The soleus muscles and EDL muscles from both legs were quickly excised under the microscope after the sacrifice. The protocol for muscle contractile function was adapted from our previous studies (Lee et al. 2015; Himori et al. 2017). During the excision procedure and force measurements, the muscles were kept in a Tyrode solution containing (in mmol/L): 121 NaCl, 5 KCl, 1.8 CaCl_2_, 0.4 NaH_2_PO_4_, 0.5 MgCl_2_, 24 NaHCO_3_, 0.1 EDTA, and 5.5 glucose. The Tyrode solution gassed with 95% O_2_‐5% CO_2_, giving a bath pH of 7.4. The proximal and distal tendons of both soleus and EDL muscles were tied with nylon thread. Muscles were mounted between a force transducer and an adjustable holder (World Precision Instruments) in a 15 mL stimulation chamber. The chamber temperature was set at 31°C with a water‐jacketed circulation bath and the muscles were bathed in the Tyrode solution continuously gassed with 95% O_2_‐5% CO_2_. A chamber temperature set at 31°C was based on the report that the inner temperature of the foot muscle yielded a value of 30.5 ± 0.5°C immediately after neck disarticulation (Bruton et al. 1998). Furthermore, 31°C is within the range of temperatures, where both muscle tetanic force and muscle endurance reach maximal capacity in mammals (Petrofsky and Lind 1981; Ranatunga and Wylie 1983; Blomstrand et al. 1985). Muscles were stimulated with supramaximal current pulses (0.5 msec duration; 150% of current required for maximum force response) via plate electrodes lying parallel to the muscles. Muscles were set to the length at which tetanic force was maximum (optimal length L_0_) and were then allowed to recover for 15 min. L_0_ was measured with a caliper and recorded. The force‐frequency relationship was determined using the following stimulus frequencies: 1 (twitch stimulus), 10, 15, 20, 30, 50, 70, 100, and 120 Hz for soleus muscles (1000 msec tetanic duration); 1, 20, 30, 40, 50, 70, 100, 120, and 150 Hz for EDL muscles (300 msec tetanic duration). At least 1 min of recovery separated electrical stimulations. After the force‐frequency protocol, skeletal muscles underwent a fatigue protocol consisting of 100 tetanic contractions for soleus muscles (70 Hz, 600 msec train duration, 2 sec interval duration) or 50 tetanic contractions for EDL muscles (100 Hz, 300 msec train duration, 2 sec interval duration). For the recovery protocol, muscle force was determined 1, 2, 5, and 10 min after the last tetanic contraction of the fatigue protocol, using the same stimulation frequency and train duration as described for the fatigue protocol. Electrically stimulated force production was expressed as absolute force (mN) and as specific force (kN/m^2^). Muscle cross‐sectional area (CSA) was assessed by dividing muscle mass by the product of muscle length and muscle density (1.06 g/cm^3^). The muscle mass was determined after the experiments by cutting the major part of the tendons.

### Statistical analysis

Data are expressed as mean ± SEM. All data were analyzed with GraphPad Prism 6 (GraphPad software Inc, La Jolla, CA). Normal distribution was checked using D'Agostino‐Pearson Omnibus normality test. Student's unpaired t‐tests or 1‐way ANOVA were used to determine significant differences in the body mass, skeletal muscle mass, estimated muscle cross‐sectional area, optimal length and peak force. When data were not normally distributed, a nonparametric t‐test (Mann–Whitney test) or a Kruskal–Wallis test was used to determine significant differences for these variables. Two‐way repeated measures ANOVA were used to analyze the force‐frequency relationship, the fatigue experiments and the recovery experiments. When appropriate, Dunnett's post hoc tests were employed. The level of significance was set at *P *<* *0.05.

## Results

### Systemic effects of docetaxel on body mass and muscle mass

**Figure 1:**
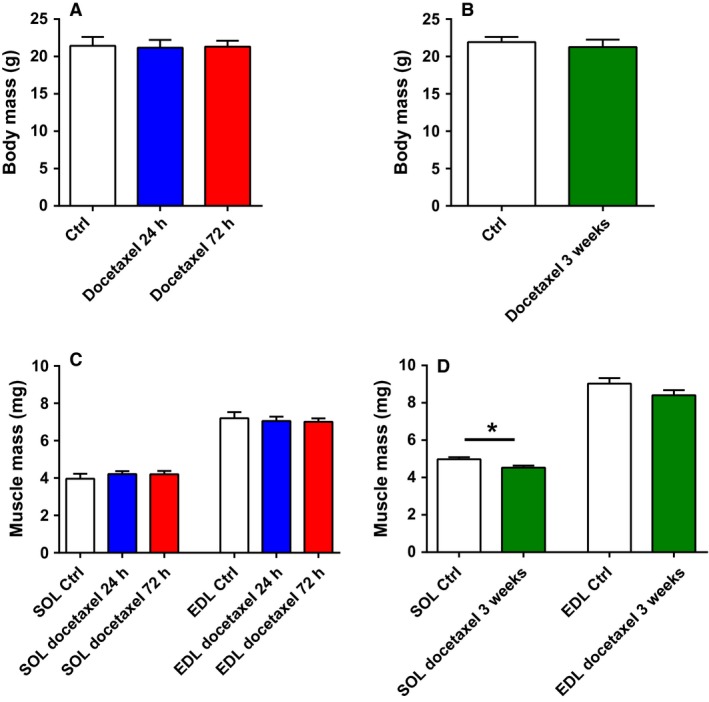
Body mass and muscle mass from healthy mice treated with docetaxel. Body mass after an acute (A; *N* = 4 animals) or a repeated (B; *N* = 6 animals) treatment. Soleus and EDL muscle masses after an acute (C; *n* = 7–8 muscles) or a repeated (D; *n* = 9–12 muscles) treatment. Ctrl: control; SOL: Soleus muscle; EDL: extensor digitorum longus muscle; *P <* *0.05.

The body mass of the mice was not significantly altered by acute or repeated docetaxel treatment (body mass 1–3% lower in treated than Ctrl mice; Fig. 1A and B). Soleus and EDL muscle masses remained unchanged 24 h and 72 h after a single injection of docetaxel (Fig. 1C). In addition, the estimated muscle cross‐sectional area was similar in all groups for both soleus muscles (Ctrl 0.31 ± 0.02 mm^2^, Docetaxel 24 h 0.35 ± 0.01 mm^2^, Docetaxel 72 h 0.35 ± 0.01 mm^2^) and EDL muscles (Ctrl 0.50 ± 0.02 mm^2^, Docetaxel 24 h 0.51 ± 0.01 mm^2^, Docetaxel 72 h 0.50 ± 0.02 mm^2^). The optimal length L_0_ was also similar in all groups for both soleus muscles (Ctrl 11.9 ± 0.1 mm, Docetaxel 24 h 11.4 ± 0.2 mm, Docetaxel 72 h 11.4 ± 0.2 mm) and EDL muscles (Ctrl 13.5 ± 0.1 mm, Docetaxel 24 h 13.1 ± 0.4 mm, Docetaxel 72 h 13.3 ± 0.2 mm).

After repeated docetaxel treatment, soleus muscle mass was ~9% lower than in the control condition (*P *<* *0.05) while only a tendency for a decreased mass in EDL muscles was observed (7%, *P *=* *0.13) (Fig. 1D). The estimated muscle cross‐sectional area was slightly lower in mice treated 3 weeks with docetaxel when compared with control animals for soleus muscles (0.46 ± 0.01 mm^2^ and 0.49 ± 0.01 mm^2^, respectively) and EDL muscles (0.61 ± 0.02 mm^2^ and 0.65 ± 0.02 mm^2^, respectively), but differences were not significant. The optimal length L_0_ was similar in both groups for soleus muscles (Ctrl 9.6 ± 0.1 mm, Docetaxel 3 week 9.3 ± 0.1 mm) and EDL muscles (Ctrl 13.2 ± 0.1 mm, Docetaxel 3 week 13.1 ± 0.1 mm). Repeated docetaxel treatment also decreased muscle mass by ~10% in tibialis anterior muscles (Ctrl 46.9 ± 0.7 mg vs. Docetaxel 3 week 42.8 ± 1.6 mg; *P *<* *0.05) and gastrocnemius muscles (Ctrl 113.2 ± 2.1 mg vs. Docetaxel 3 week 100.1 ± 3.4 mg; *P *<* *0.01).

### Muscle function following acute docetaxel treatment

**Figure 2:**
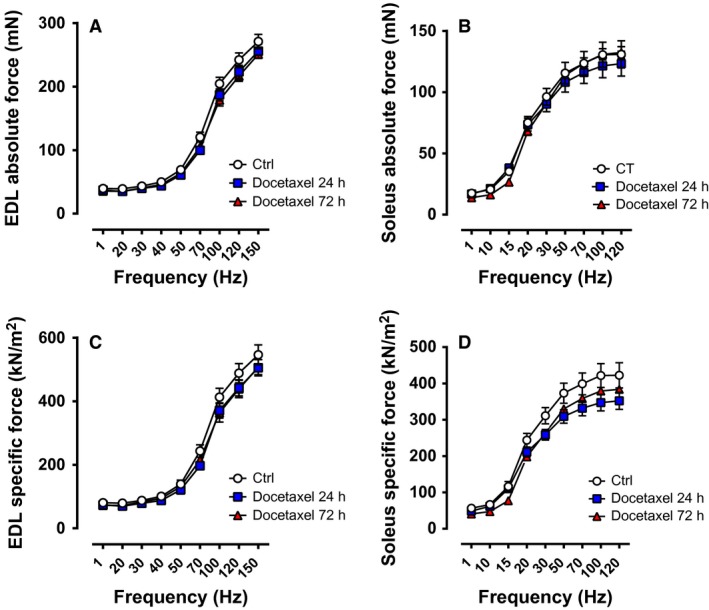
Force‐frequency relationship of muscles from healthy mice treated acutely with docetaxel. Absolute force of EDL muscles (A; *n* = 8 muscles) and soleus muscles (B; *n* = 7–8 muscles). Specific force of EDL muscles (C; *n* = 8 muscles) and soleus muscles (D; *n* = 7–8 muscles). Ctrl: control. The force‐frequency relationships were determined at 1, 20, 30, 40, 50, 70, 100, 120, and 150 Hz for EDL muscles (300 ms tetanic duration), and at 1, 10, 15, 20, 30, 50, 70, 100, and 120 Hz for soleus muscles (1000 ms tetanic duration).

Figure 2 presents the absolute and specific force‐frequency relationship in EDL and soleus muscles from mice administered with a single IV injection of docetaxel (Docetaxel 24 h, Docetaxel 72 h). The absolute force was similar between groups (Ctrl, Docetaxel 24 h, and Docetaxel 72 h) in both EDL and soleus muscles (Fig. 2A and B). When normalized to cross‐sectional area, specific force remained unchanged between the experimental groups for the EDL muscles (Fig. 2C).

According to the two‐way repeated measures ANOVA, there were no significant global effect of docetaxel on the specific force of soleus muscles (Fig. 2D; *P *=* *0.15). To evaluate whether acute chemotherapy treatment specifically affected the peak specific force of soleus muscles, a one‐way ANOVA was performed at 120 Hz. The peak specific force of soleus muscles was 17% lower in the Docetaxel 24 h group than in the Ctrl group, but the differences did not reach the level of significance (*P *=* *0.13).

**Figure 3:**
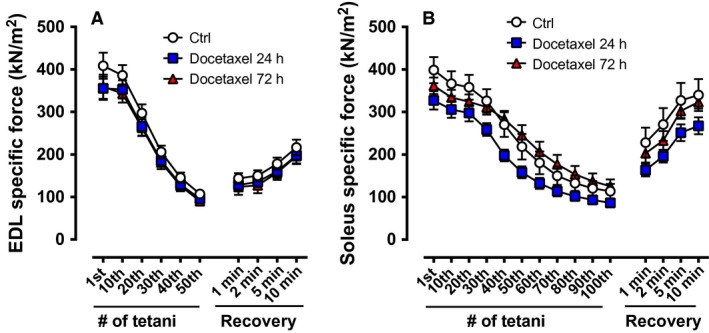
Specific muscle force during repeated electrical stimulations and during recovery after repeated contractions from healthy mice treated acutely with docetaxel. Specific force of EDL muscles during 50 tetanic contractions (100 Hz, 300 ms train duration, 2 s interval duration) and after 1, 2, 5, and 10 min following repeated contractions (A; *n* = 8 muscles). Specific force of soleus muscles during 100 tetanic contractions (70 Hz, 600 ms train duration, 2 s interval duration) and after 1, 2, 5, and 10 min following repeated contractions (**B**;* n* = 7–8 muscles). Ctrl: control.

Figure 3 depicts the specific force during repeated electrical stimulations and during recovery after repeated contractions. Ex vivo experiments have demonstrated that whole soleus muscles display a larger decrease in force production during fatiguing stimulation than single soleus fibers (Zhang et al. 2006). This accelerated force decline in whole muscle during repeated contractions has been attributed to the limited O_2_ diffusion from the surface of the muscle, thereby leading to a reduced O_2_ delivery to muscle fibers and the presence of hypoxia (Zhang et al. 2006). We were aware of this limitation, but still wanted to test whether whole muscles from mice treated or not with docetaxel respond differently to increased metabolic stress induced by repeated stimulations and during recovery after repeated contractions. The decline in specific force during the repeated contractions and the force recovery after the fatiguing protocol were similar between groups for the EDL muscles (Fig. 3A). The soleus specific force was slightly lower in the Docetaxel 24 h group compared with the Ctrl group during the repeated contractions and the recovery experiment, but the differences were not significant (Fig. 3B, main effect of chemotherapy *P *=* *0.09 from the two‐way repeated measures ANOVA).

### Contractile function in skeletal muscle following repeated docetaxel treatment

**Figure 4:**
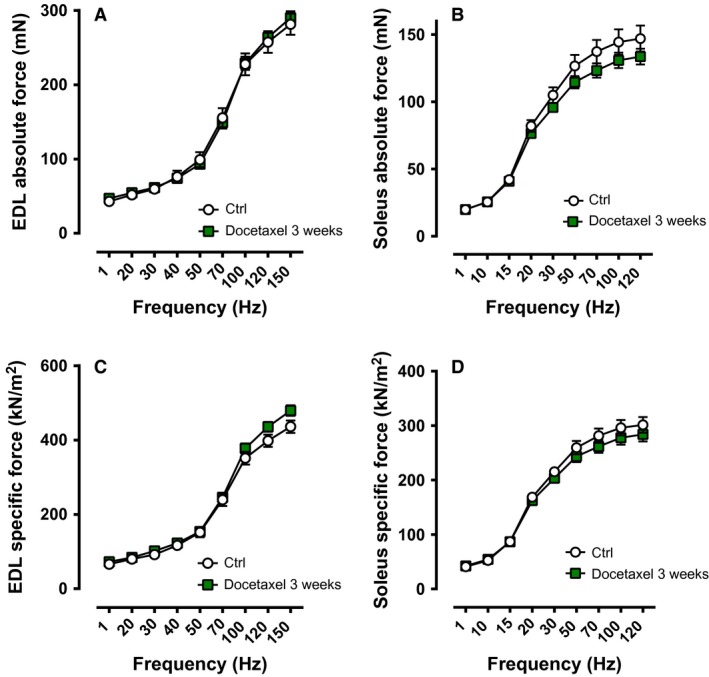
Force‐frequency relationship of muscles from healthy mice treated repetitively with docetaxel (3‐week treatment). Absolute force of EDL muscles (**A**;* n* = 10–11 muscles) and soleus muscles (**B**;* n* = 9–12 muscles). Specific force of EDL muscles (**C**;* n* = 10–11 muscles) and soleus muscles (**D**;* n* = 9–12 muscles). Ctrl: control. The force‐frequency relationships were determined at 1, 20, 30, 40, 50, 70, 100, 120, and 150 Hz for EDL muscles (300 ms tetanic duration), and at 1, 10, 15, 20, 30, 50, 70, 100, and 120 Hz for soleus muscles (1000 ms tetanic duration).

**Figure 5:**
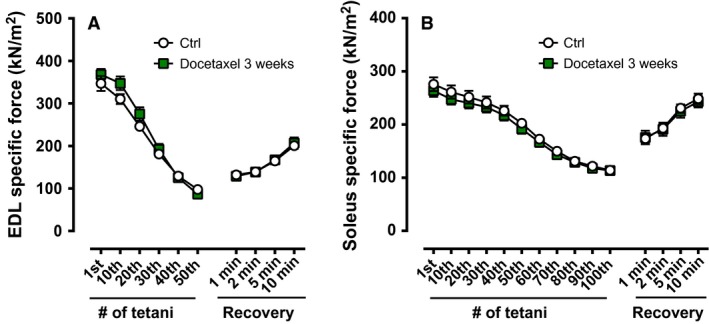
Specific muscle force during repeated electrical stimulations and during recovery after repeated contractions from healthy mice treated repetitively with docetaxel (3‐week treatment). Specific force of EDL muscles during 50 tetanic contractions (100 Hz, 300 ms train duration, 2 s interval duration) and after 1, 2, 5, and 10 min following repeated contractions (**A**;* n* = 10–11 muscles). Specific force of soleus muscles during 100 tetanic contractions (70 Hz, 600 ms train duration, 2 s interval duration) and after 1, 2, 5 and 10 min following repeated contractions (**B**;* n* = 9–12 muscles). Ctrl: control.

Similar to one dose of docetaxel (24 h, 72 h), the absolute and specific force in both EDL and soleus muscles were not altered after the 3 weeks of treatment as compared to the control group (Fig. 4). In addition, soleus and EDL muscles from the 3 week docetaxel treatment did not respond any differently than the controls to repeated contractions or during the recovery phase (Fig. 5).

## Discussion

It is well established that patients with cancer display decreased physical fitness, reduced muscle strength and increased fatigue (Curt et al. 2000; Santiago‐Palma and Payne 2001; Perry et al. 2007). Many factors have been proposed to explain cancer‐related muscle dysfunction, including tumor‐derived factors, physical inactivity, malnutrition, surgery, and cancer treatments (Christensen et al. 2014). In particular, decreased physical function, fatigue and muscle weakness have been observed in patients with cancer undergoing chemotherapy (Stone et al. 1999; Harrington et al. 2011). To understand whether chemotherapy *per se* affects skeletal muscle function, we here treated healthy mice acutely or repetitively with docetaxel (20 mg/kg). In contrast to our hypothesis, we demonstrate that chemotherapeutic treatment using docetaxel does not significantly impair muscle force production in healthy mice.

A loss of body mass and muscle wasting have been observed in cancer patients undergoing chemotherapy (Koskelo et al. 1990), but the results remain controversial (Ida et al. 2014; Jeon et al. 2014). Body composition and muscle mass are influenced by multiple factors in cancer patients, including the type and disease stage of cancer, the specificity of the treatment/intervention (surgery, radiation, chemotherapy, etc.), and personal habits (physical activity, nutritional status, etc.) (Freedman et al. 2004; Fuchs‐Tarlovsky et al. 2013; Ryan et al. 2016; Wall et al. 2017). Here, we show that docetaxel *per se* (one single injection or weekly injections for 3 weeks) does not decrease body mass in healthy mice. A loss of body mass has been previously seen in healthy mice treated with docetaxel during a short period of time (3 and 5 days) (Wang et al. 2015). However, in this study, the drug (20 mg/kg) was injected intraperitoneally for consecutive days (2 or 4 injections), which makes the comparison to our study difficult. A loss of body mass has also been observed in healthy mice treated with doxorubicin, with changes observed after both a single IP injection (Gilliam et al., 2009, Gilliam et al. 2016) and a single IV injection (Gilliam et al. 2011a,b). Furthermore, we observed that 3 weeks of docetaxel treatment slightly reduces the mass of hind limb muscles (~10%), whereas a more severe reduction in muscle mass (17–39%) has been observed after a short period of consecutive IP injections (2 or 4 injections) with this drug in mice (Wang et al. 2015). Our findings imply that intravenous administration of docetaxel during long‐term treatment induces only minor muscle atrophy without affecting body weight in healthy mice. To date, it remains to be clarified which are the main chemotherapy‐related factors (type of chemotherapy drugs, mode of injection, number of injections, duration of treatment, etc.) responsible for the loss of body weight and muscle mass in mouse and human.

Here, we show that acute or repeated docetaxel treatment does not severely reduce muscle force production in healthy mice. A slight, but nonsignificant decrease in force production was only observed in the soleus muscles 24 h after a single injection of docetaxel (peak specific force, specific force during the repeated contractions and during the recovery experiment). Several studies have demonstrated that acute or repeated treatment with doxorubicin, an anthracycline antibiotic used as a chemotherapy drug to treat malignant cancer, impairs muscle function in healthy mice (Gilliam et al., 2009, Gilliam et al. 2011a,b; Gilliam and St Clair 2011; Gilliam et al. 2016; Ertunc et al. 2009). In particular, a single IP injection with doxorubicin (20 mg/kg; force measurements performed 72 h later) reduced the peak specific force by ~35% in diaphragm muscles (Gilliam and St Clair 2011; Gilliam et al. 2011a), while the changes were of lower magnitude in hind limb muscles, including the EDL muscles (~28% decrease) (Gilliam et al., 2009) and the soleus muscles (~24% decrease or no differences) (Gilliam and St Clair 2011; Gilliam et al. 2016). Interestingly, Gilliam et al. (2011a) examined whether doxorubicin‐induced muscle weakness was influenced by the mode of injection (IV or IP, 20 mg/kg) (Gilliam and St Clair 2011).They showed that peak specific force of soleus and EDL muscles was not impaired 3 days following a single IV injection of doxorubicin (Gilliam and St Clair 2011). Furthermore, they concluded that an IP injection exacerbates diaphragm weakness compared with an IV injection (Gilliam et al. 2011a). In the latter study, local inflammation and sarcolemmal disruption were observed on the abdominal surface of the diaphragm muscle in response to an IP injection, but not an IV injection of doxorubicin (Gilliam et al. 2011a). This finding suggests that injection of this drug into the peritoneal cavity induces peritonitis, thereby leading to abdominal and systemic inflammation, which ultimately could promote muscle weakness. Nevertheless, further studies clarifying how the mode of injection affects muscle force production would be valuable.

Although IV injection/s of docetaxel has/have no clear impact on force production of hind limb muscles in healthy mice, further experiments are needed to evaluate whether this type of chemotherapy treatment will have similar effects on mice with cancer. One possibility is that docetaxel administration will exacerbate muscle dysfunction in cancer models associated with severe muscle weakness and atrophy, such as models of lung cancer, colon cancer or breast cancer (Choi et al. 2013; Norden et al. 2015; Waning et al. 2015). In addition, our work cannot exclude the possibility that IV injection of docetaxel has a negative effect on muscle function in humans, leading to accentuation of muscle dysfunction in patients with cancer. Furthermore, due to its cytotoxic effects, docetaxel can also induce neurosensory disturbances and peripheral neuropathy in patients with cancer, which can be persistent (Eckhoff et al. 2015) and promote side effects such as muscle and joint pain (Eckhoff et al. 2013). Thus, it is possible that docetaxel chemotherapy in cancer patients increases central fatigue and/or induces neuropathy, which then result in decreased physical fitness, muscle weakness and fatigue.

In conclusion, this study shows that acute or repeated chemotherapy treatment with IV injection of docetaxel does not impair muscle force production in healthy mice. The controversy with the literature is certainly due to the mode of injection (IP or IV) and the type of drugs administrated in mice (docetaxel or doxorubicin). Furthermore, it remains to be elucidated whether; (1) the cancer itself (2) and/or its combination with docetaxel (or other chemotherapy drugs) directly affect muscle function in patients and whether (3) the side effects associated with chemotherapy treatment (neurotoxicity, central fatigue, decreased physical activity, etc.) result in muscle weakness and fatigue.

## Conflict of Interest

None declared.
